# Whole exome sequencing reveals a novel LRBA mutation and clonal hematopoiesis in a common variable immunodeficiency patient presented with hemophagocytic lymphohistiocytosis

**DOI:** 10.1186/s40164-021-00229-y

**Published:** 2021-06-13

**Authors:** Yanling Ren, Feng Xiao, Fei Cheng, Xin Huang, Jianhu Li, Xiaogang Wang, Wei Lang, Xinping Zhou, Jianping Lan, Hongyan Tong

**Affiliations:** 1 Myelodysplastic Syndromes Diagnosis and Therapy Center, Department of Hematology, The First Affiliated Hospital, College of Medicine, Zhejiang University, 79# Qingchun Road, Hangzhou, 310003 Zhejiang China; 2 Department of Hematology, The First Affiliated Hospital, College of Medicine, Zhejiang University, Hangzhou, 310003 Zhejiang China; 3 Department of Pathology, The First Affiliated Hospital, College of Medicine, Zhejiang University, Hangzhou, 310003 China; 4 Department of Hematology and Hematopoietic Stem Cell Transplant Center, Zhejiang Provincial People’s Hospital, Hangzhou, 310014 Zhejiang China

**Keywords:** Common variable immunodeficiency, LPS-responsive beige-like anchor, Hemophagocytic lymphohistiocytosis, Myeloid malignancy, Hematopoietic stem cell transplantation

## Abstract

Common variable immunodeficiency (CVID) was a kind of primary immunodeficiency disorders with heterogeneous phenotype and genotype. Lipopolysaccharide-responsive and beige-like anchor (LRBA) mutation was identified as disease associated in CVID, advanced genetic method will help to detect atypical cases. We report a case of adult patient manifested as hemophagocytic lymphohistiocytosis (HLH), bone marrow examination suggested prosperity to MDS, manifested as increased immature myeloid cells and dysplastic hematopoiesis. Whole exome sequencing (WES) identified a novel heterogeneous c.1876T > C (p.W626R) mutation in *LRBA* and four somatic mutations: *ASXL1* (c.1967dupA); *PTPN11* (c.226G > A), *U2AF1* (c.101C > T and c.470A > G), among which *ASXL1* was a high-risk marker of clonal hematopoiesis. Combined with her recurrent severe infections and immune abnormalities such as hypoimmunoglobulinemia, the patient was diagnosed with CVID. Subsequent hematopoietic stem cell transplantation saved her from severe cytopenia and immune deficiency. This case report highlights the great promise of utilization of WES for diagnosing rare disease with atypical manifestations and guiding further treatment.

**Supplementary Information:**

The online version contains supplementary material available at 10.1186/s40164-021-00229-y.

Letter to the Editor,

Hemophagocytic lymphohistiocytosis (HLH) is a rapidly progressing and highly fatal disease, and the prognosis is closely related to the treatment of the primary disease [1], so it is particularly important to actively search for the cause while treating HLH. Common variable immunodeficiency (CVID) is the most prevalent primary immunodeficiency disorder with heterogeneous phenotype and genotype [2], a timely and accurate diagnosis is also urgent to prevent significant morbidity and mortality [3]. Here, we report an adult CVID patient manifested as HLH, whole exome sequencing (WES) revealed LPS-responsive beige-like anchor (LRBA) and myeloid malignancy associated mutations may be the genetic cause. Finally, hematopoietic stem cell transplantation (HSCT) saved her from severe cytopenia and immune deficiency.

A 46 years old female patient who presented with fever and fatigue as well as cytopenia for 20 days was referred to our center, physical examinations showed significantly hepatosplenomegaly. Laboratory tests revealed low fibrinogen and elevated ferritin and soluble CD25. Bone marrow smear showed an increase of phagocytosis (Fig 1A). Flow cytometry showed increased abnormal granulocytes of 76.45% (Fig 1B), while biopsy showed proliferative hematopoiesis with dysplastic erythroid and increased immature cells (Fig 1C). Hypogammaglobinemia and abnormal T cell subpopulations of peripheral blood were also detected (Table 1). Image test suggested pneumonia but no evidence of malignancy (Additional file 1: Figure S1). Therefore, the diagnosis of HLH is established. Viral infections were excluded by negative serology results. Her previous history showed recurrent respiratory tract infection, severe human papilloma virus infection led to total hysterectomy and severe virus pneumonia which needed ventilator adjuvant treatment. Unexplained hepatosplenomegaly and hypogammaglobinemia was found then, but no severe cytopenia, thus CVID was established. WES analysis detected a novel single heterozygous mutation of *LRBA* (c.1876T > C; p.W626R). Another 4 somatic mutations which suggested clonal hematopoiesis were also identified: *ASXL1*(c.1967dupA); *PTPN11*(c.226G > A); *U2AF1*(c.101C > T and c.470A > G). After obtaining the informed consent, the same mutation of *LRBA* in her healthy father, little brother and her son were confirmed by Sanger sequencing, but no such mutation in her mother and the older brother (Fig 1D).

**Fig. 1:**
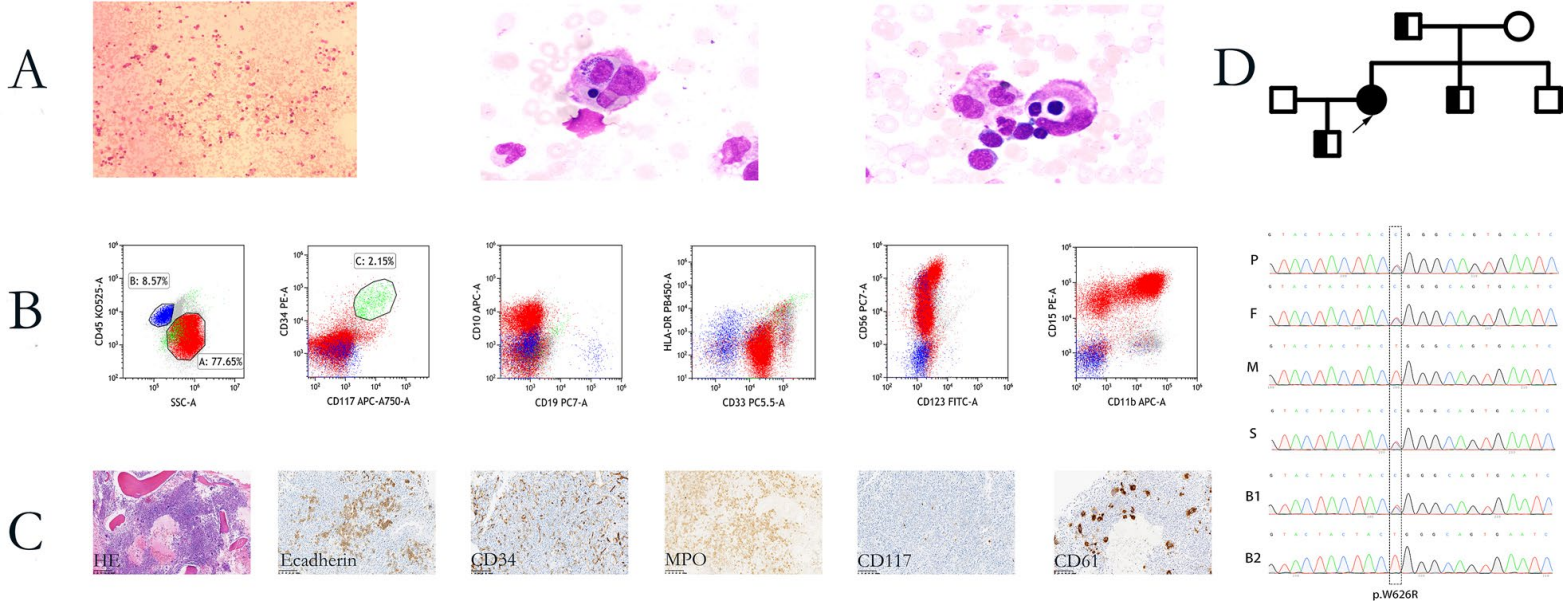
Bone marrow features: **A** Phagocytosis of red cells and platelets by hemophagocytichistiocytes; **B** Abnormal granulocytes and blast by flow cytometry; **C** Immunochemistry of BM specimen suggested an increase in immature myeloid cells which are positive for CD33, CD117, CD45-SSA, and CD11B; **D** Pedigree of the family and Sanger sequencing confirming the mutation status of LRBA in the patient and her parent, brother and son (F, father; M, mother; Patient; S, Son; B1, brother 1; B2,brother 2)

**Table 1:** Immunological parameters

Parameters	Measurement at diagnosis	Measurement after HSCT (4 months)	Normal range
Hemoglobin, g/L	57	110	115–150
Platlet,×10^9^/L	5	133	100–300
Leucocytes, ×10^9^/L	7.5	4.5	4–9
Lymphocytes, ×10^9^/L	0.67	1.8	1.1–3.2
B cells, %	2.8	7.26	5.1–20.3
T cells	95.2	86.74	53.7–80.9
CD3^+^CD4^+^, %	19.9	14.8	19.57–48.02
CD3^+^CD8^+^, %	75.3	71.94	15.8–37.5
CD4^+^CD25^+^FOXP3^+^,%	0.23	0.49	1.11–2.9
NK cell, %	2.0	6	6.7–30.9
Total T/NK lymphocyte, %	8.96	16.94	17.06–43.35
Cytotoxic T/NK Lymphocyte,%	2.29	3.73	2.86–18.22
Non-Cytotoxic T/NK Lymphocyte,%	6.68	13.21	11.15–35.76
IGG, mg/dL	543	1160	860–1740
IGA, mg/dL	10	79	100–420
IGM, mg/dL	18	123	50–280
C3, mg/dL	78	124	70–140
C4, mg/dL	19	36	10–40
sCd25, pg/ml	22914	5367	< 6400
NK cytotoxicity	22.96%	17.44%	≥ 15.11%

According to the HLH-2004 protocol, glucocorticoid and etoposide was given to her. Her fever improved and the spleen was significantly shrinked, but she still had cytopenia. On July 14th 2020, she underwent haploindentical allogeneic HSCT from her older brother. Until the time of writing this article, the patient showed normal blood cell count, close to normal immunoglobulin except for low IGA, no mutations were detected for another WES analysis.

Homozygous and compound heterozygous mutations of *LRBA* were identified as one cause of CVID recently, which could decrease or abolish LRBA protein expression, thus resulting in very low cytotoxic T lymphocyte–associated antigen 4 (CTLA4) expression and dysfunction of T cells [4]. Some researchers believed that mild phenotypes were associated with compound heterozygous mutations and residual protein expression of LRBA, while other research showed that LRBA protein expression levels are not correlated with clinical phenotypes [5, 6].Unfortunately, we have not tested the protein expression of LRBA in time, the T-to-C transition at position c.1876 in exon 14 of *LRBA* caused a substitution at position p. W626R(Cosmic 8925453), which was not in the main functional structural regions of the protein and we speculated that the novel mutation may be associated with LRBA structural instability and damaged regulatory function, just as reported by Pauline A et al., monoallelic LRBA mutation was disease associated [7]. Besides, possible polygenic and epigenetic factors could also involve in CVID pathogenesis, including *PTPN11 *[8, 9]. Just as in our case, mutations of *ASXL1*, *U2AF1* and *PTPN11* combined with *LRBA* may participate in the formation of clinical phenotype. Importantly, CVID patients had a higher risk of developing malignancies, mainly lymphoma, MDS were rarely reported [10]. Increased immature cells and dysplastic hematopoiesis as well as the detection of clonal marker such as mutations in *ASXL1* and *U2AF* all suggested the diagnosis for MDS [11]. Moreover, after HLH had been effectively controlled, cytopenia was not improved, all prompting us to perform HSCT as soon as possible.

In conclusion, we report a rare case of patient with CVID manifested as HLH had *LRBA* mutation and clonal hematopoiesis. WES played an important role in etiology diagnosis and guiding treatment, thus should be considered in patients with atypical manifestations.

## Supplementary Information

**Additional file 1: Figure S1.** **A** Scattered inflammation of both lungs and pleural effusion by chest CT scan. **B** PET-CT demonstrated enlargement of the spleen, mild increased FDG in the bone marrow and sinusitis.

## Data Availability

The single institute data from this study is available from the corresponding author upon reasonable request.
