# Supplementary figures and images for: Whole exome sequencing reveals a novel LRBA mutation and clonal hematopoiesis in a common variable immunodeficiency patient presented with hemophagocytic lymphohistiocytosis
Source: Exp Hematol Oncol. 2021 Jun 13;10:38. doi: 10.1186/s40164-021-00229-y (PMC8201866; doi:10.1186/s40164-021-00229-y)

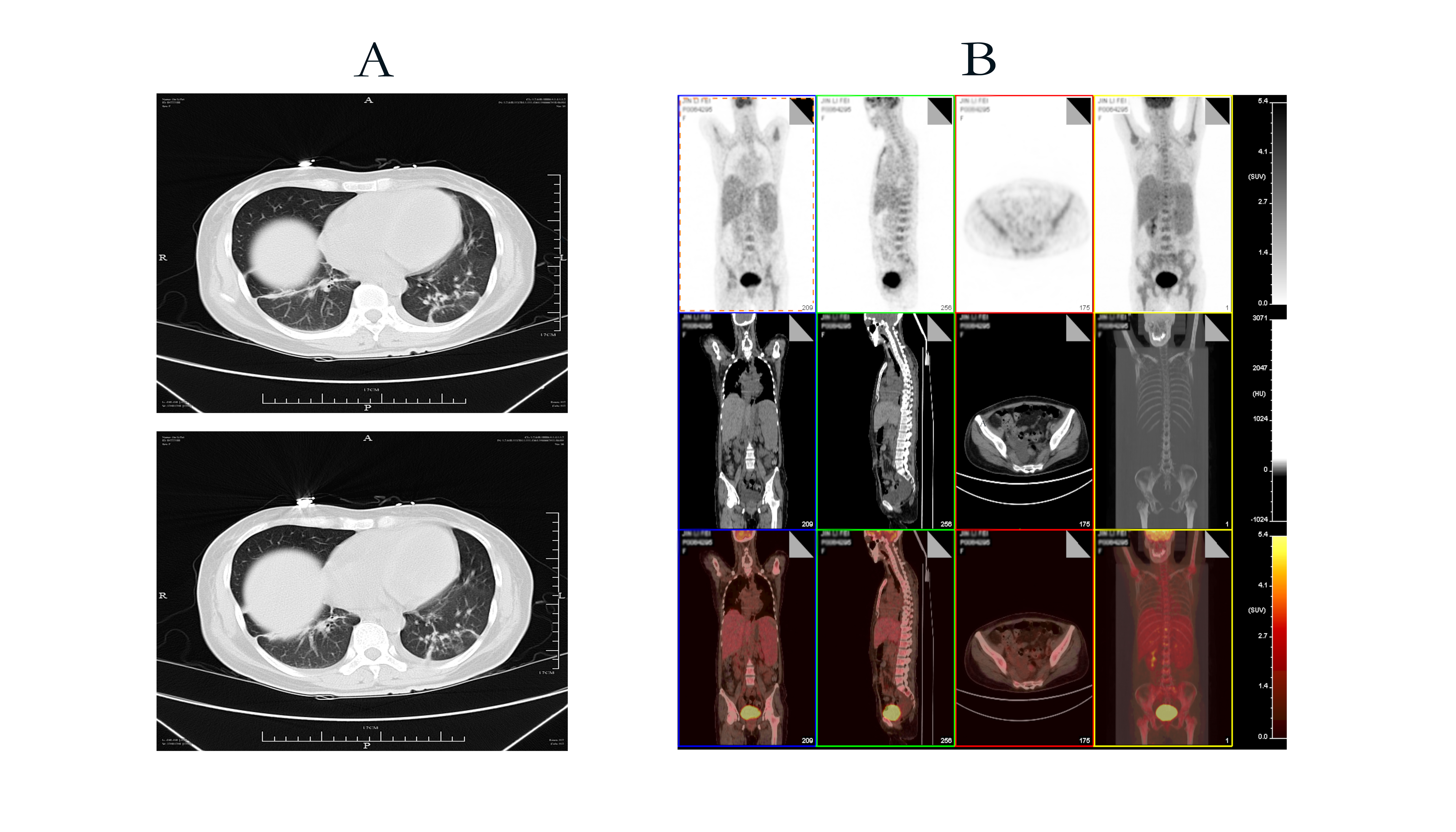

Supplement: Supplementary file 1 — Additional file 1: Figure S1. A Scattered inflammation of both lungs and pleural effusion by chest CT scan. B PET-CT demonstrated enlargement of the spleen, mild increased FDG in the bone marrow and sinusitis. [file 40164_2021_229_MOESM1_ESM.jpg]
